# The Human Omental Adipose Depot Mitigates Inflammation, Immune Response, and Oxidative Stress Pathways in Response to Injury via Its Secretome

**DOI:** 10.3390/biology14111509

**Published:** 2025-10-28

**Authors:** Meredith Krause-Hauch, Rekha S. Patel, Bangmei Wang, Brianna Jones, Paul Albear, Niketa A. Patel

**Affiliations:** 1 James A. Haley Veteran’s Hospital, 13000 Bruce B. Downs Blvd, Tampa, FL 33612, USA; meredith.krause-hauch@va.gov (M.K.-H.); rekha.patel1@va.gov (R.S.P.); bangmei.wang@va.gov (B.W.); paul.albear@va.gov (P.A.); 2 Department of Chemistry, University of South Florida, Tampa, FL 33620, USA; bmjones1@usf.edu; 3 Department of Molecular Medicine, University of South Florida, Tampa, FL 33620, USA

**Keywords:** exosomes, omentum, adipose stem cells, RNAseq, inflammation, oxidative stress, immune response

## Abstract

**Simple Summary:**

In humans, there is a fat layer called the omentum that surrounds the internal abdominal organs such as the stomach and pancreas. Omental fat (omental adipose tissue) is shown to be beneficial to prevent infections at the surgical site. Though this method of transposing omental fat to the top of surgical incisions is tested in clinics, the mechanism by which omental fat promotes repair is not well known. Here, we demonstrate that omental adipose tissue contains stem cells, and we characterize them in the laboratory. We show that human omental adipose stem cells (om-hASCs) secrete nano-sized vesicles (circular sacs) called exosomes that contain cargo such as proteins. We studied the properties of om-hASC exosomes (om-hASCexos) and subsequently used injury models in cell culture as well as rodent wound models to evaluate their function. Our results demonstrate that om-hASCexos significantly accelerate the healing of wounds. Using next-generation sequencing, called RNAseq, we identified the genes and pathways involved in the immune response and inflammation that were positively affected by om-hASCexo treatment. Overall, we demonstrate that om-hASCexos have unique properties that shed light on how the body responds to internal injuries and insults such as ruptured ovaries, inflamed appendices, or ulcerated intestines.

**Abstract:**

Human intraperitoneal omental adipose tissue, part of the visceral adipose depots, surrounds the abdominal organs and has functions distinct from the subcutaneous adipose depots. In the clinical setting, it is observed that the omentum is beneficial to combat internal sources of inflammation, oxidative stress, and injury-related stress. However, the molecular mechanisms involved in these functions are not fully understood. We previously demonstrated that adipose stem cells derived from human omental adipose tissue (om-hASCs) secrete exosomes (exos). We and others have extensively evaluated the subcutaneous adipose depot-derived exosomes; however, the role of adipose stem cells derived from the human omental depot (om-hASCs) remains less known. In this study, we postulated that exosomes from om-hASCs (om-hASCexos) drive the repair ability of the omentum to heal organs after internal injury and insults. First, we characterized the om-hASCexos using a proteomic analysis which identified the distinct cargo. Using in vitro injury models, we show that om-hASCexos significantly improve cell migration and proliferation, while decreasing oxidative stress and inflammation. To study acute in vivo healing, a rat wound model was evaluated. Om-hASCexos significantly improved the healing rate of injuries. RNAseq revealed that om-hASCexo treatment acts upon pathways associated with lipid and fatty acid metabolism, apoptosis, immune response, and cell differentiation. The pathway analysis indicated that om-hASCexos significantly regulate the expression of *Clec5a* and *Trem1* in the immune response pathway. Overall, we demonstrate the singular properties of om-hASCexos that are distinct from other sources of hASC. Thus, this study provides an understanding of the unique ability of the omental adipose depot to combat internal injuries.

## 1. Introduction

White adipose tissue depots (i.e., subcutaneous and visceral depots) store energy and are also endocrine organs secreting hormones and cytokines. While the subcutaneous depot is located just under the skin, allowing for easy access, the visceral depot is located deeper in the abdomen, surrounding organs such as the stomach and pancreas. The mesenteric and omental adipose depots, which are the intraperitoneal components of the visceral adipose depot, drain into the hepatic portal vein. The omentum is known to respond to inflammation by secreting angiogenic factors and growth factors such as b-FGF and VEGF [1]. It is also proposed that the omentum may move throughout the peritoneal cavity to control infection, inflammation, revascularization, and tissue regeneration [2,3]. The omentum is highly immunogenic and contains lymphoid aggregates called milky spots. During times of inflammation within the peritoneal cavity, the omentum functions in the absorption of bacteria and the production of leukocytes [3] and facilitates the isolation of internal injuries or inflamed areas from the rest of the body [4]. The omentum has been known to respond to internal wounds or injuries, including surgical wounds, ruptured ovaries, inflamed appendices, and ulcerated intestines [4]. Under certain circumstances, surgeons have used omental transposition around surgical sites due to the high vascularization and infection prevention properties of the omentum [5,6,7]. Although its anti-inflammatory and regenerative properties are well documented in the clinic, the mechanism of action underlying the ability of omental adipose tissue to heal wounds is less known.

We previously demonstrated that human mesenchymal stem cells can be derived from omental adipose tissue (om-hASCs) and characterized its stem cell antigens and markers [8]. Cell-to-cell communication can occur via the secretion of extracellular vesicles (EVs) [9], which communicate with other cells via surface proteins and cargo molecules [10]. EVs are generally classified as apoptotic bodies, micro vesicles, or exosomes [11]. EVs are utilized by most types of cells for the transfer of proteins, lipids, and nucleic acids. This transfer of cargo between cells results in EV participation in biological processes such as cell motility, differentiation, proliferation, apoptosis, and immunity [12]. Exosomes are the smallest EVs, at ~40–160 nm in diameter [13]. Their small size as well as their high stability make them ideal carriers of endogenous cargo to respond to internal trauma [14].

Due to the known interaction of the omentum with internal injuries, here, we hypothesized that the exosomes from the secretome of omental adipose stem cells are critical to its mode of action. Hence, we first undertook a proteomic analysis of exosomes secreted from human omental adipose stem cells (om-hASCexos). Next, we investigated the effects of om-hASCexos in in vitro injury models mimicking oxidative stress and inflammation. Our results, using an in vivo wound model followed by RNAseq analysis, demonstrate that om-hASCexos affect metabolic pathways, immune response, and cytokine production, as well as cell death and differentiation. These results thus elucidate the mechanisms through which the omentum protects and promotes tissue repair post-trauma and injury and give an insight into the unique biological properties of the omental adipose depot.

## 2. Materials and Methods

### 2.1. Cell Culture

The pooled omental adipose stem cells (om-hASCs) were purchased from ZenBio, (Durham, NC, USA) and were cultured in ZenBio preadipocyte media. Human dermal fibroblasts (HDFs) (American Type Culture Collection, Manassas, VA, USA) were plated in DMEM supplemented with 10% NCF prior to any experimentation. The cells were maintained at 37 °C in a humidified 5% CO_2_ atmosphere.

### 2.2. Isolation of Exosomes from Om-hASCs

Pooled human omental adipose stem cells (om-hASCs) were purchased from ZenBio, (Durham, NC, USA) and were cultured in hASC medium (ZenBio, Cat# PM-1) until 90% confluent. Exosomes were isolated from the conditioned media of om-hASCs as follows. The om-hASCs were then cultured in serum exosome-free hASC medium for 48 h. The conditioned media (CM) was collected and centrifuged for 10 min at 1500× *g* for removal of debris. The supernatant was then centrifuged at 10,000× *g* for 10 min. The supernatant was filtered through a 0.22 μm filter. It was then concentrated using a 10 kDa molecular weight cut-off filter (MWCO) using Amicon Ultracentrifugal filters (MERCK, Rahway, NJ, USA, Cat# C7715). The concentrated supernatant was centrifuged at 14,000× *g* for 45 min. The supernatant was then applied to Size Exclusion Chromatography (SEC) using the Izon qEV 35 nm Legacy kit (Izon, Christchurch, New Zealand). This column removes lipoproteins such as LDL and HDL and separates exosome particles with high purity. PBS was freshly filtered using a 0.22 μm filter and used to flush the qEV columns prior to sample application. The filtered PBS was used to elute exosomes. Samples were collected after flowing through the qEV column using the Automated Fraction Collector (AFC) (IZON, Medford, MA, USA). Magnetic beads with CD9 (CD9 Exo-flow capture kit, System Biosciences, Palo Alto, CA, USA) were used to obtain highly purified exosomes. Exosome diameter and concentration were analyzed using NanoSight (Salisbury, UK) Nanoparticle Tracking Analysis (NTA), NTA3.1, Build 3.1.46 RRID SCR-014239.

### 2.3. Droplet Digital Polymerase Chain Reaction (ddPCR)

To determine gene expression of lncRNAs *GAS5* and *MALAT1* in the om-hASCexos compared to subcutaneous depot-derived hASCexos, ddPCR (Bio-Rad Laboratories, Hercules, CA, USA) was performed. Primers were combined with cDNA synthesized from 500 ng of RNA at a 1:100 dilution in QX200 ddPCR EvaGreen Supermix (Bio-Rad, Hercules, CA, USA). The QX200 Droplet Generator from Bio-Rad (Hercules, CA, USA) was used for droplet production. Droplets were then analyzed with the QX200 droplet reader for gene copies. The results were analyzed using QuantaSoft™ Analysis Pro version 1.7.4.0917 (Bio-Rad) in 2D amplitude mode then exported to Excel for further calculations as previously described [15].

### 2.4. Transmission Electron Microscopy (TEM)

Transmission Electron Microscopy (TEM) images were obtained to visualize the morphology of the om-hASCexos. Procedures are described in our previous publication [16]. Briefly, 4 μL of om-hASCexo preparation was placed on a carbon-filled coated copper mesh grid and incubated at room temperature for 10 min. Excess liquid was removed prior to three washes with 0.2-micron filtered, boiled distilled water to remove PBS. Samples were dried overnight, then imaged using a 1400 Transmission Electron Microscope (JEOL, Tokyo, Japan) at 100kx magnification.

### 2.5. Proteomics

To analyze the protein cargo of om-hASCexos compared to that of exosomes derived from subcutaneous adipose, we used liquid chromatography–mass spectrometry (LC–MS). We characterized peptides via a Thermo Q-exactive-HF-X mass spectrometer coupled to a Thermo Easy nLC 1200 (Thermo Fisher, Waltham, MA, USA). Samples were separated at 300 nL/min on an Acclaim PEPMAP 100 trap (75 μM, 2 cm, c18 3 μm, 100 A; Thermo Fisher, Waltham, MA, USA) and a Thermo easy spray column (75 μm, 25 cm, c18, 100 A; Thermo Fisher, Waltham, MA, USA) using a 120 min gradient with an initial starting condition of 2% buffer B (0.1% formic acid in 90% Acetonitrile) and 98% buffer A (0.1% formic acid in water). Buffer B was increased to 28% over 140 min, then to 40% over 10 min. Then, 90% Buffer B was run for 15 min. The mass spectrometer was outfitted with a Thermo nanospray easy source (Thermo Fisher, Waltham, MA, USA) with the following parameters: Spray voltage: 2.1 V, Capillary temperature: 300 dC, Funnel RF level = 40. MS data was acquired at a resolution of 60,000, AGC target of 3 × 10^6^, and a max IT time of 50 ms. The range was set to 400–1600 *m*/*z*. MS/MS data was acquired with a resolution of 15,000, an AGC of 1 × 10^4^, and max IT of 50 ms. The top 30 peaks were picked, with an isolation window of 1.6 *m*/*z* and with a dynamic execution of 25 s. Resulting samples were processed using Max Quant v 2.3.1.0. A reviewed human data base was downloaded from Uniprot and searched with the following parameters: tryptic enzyme with a max of two missed cleavages, a precursor mass tolerance of 10 ppm, and a fragment mass tolerance of 0.02 Da. Modifications included Oxidation, Acetyl, and Carbamidomethyl. FDR rate was set at 0.01 and the ratio between protein intensities was calculated.

### 2.6. In Vitro Scratch Assay

An in vitro scratch assay was used to evaluate the effect of om-hASCexo treatment on cell migration and proliferation. Human dermal fibroblasts (HDFs) (American Type Culture Collection, Manassas, VA, USA) were grown to confluency in DMEM supplemented with 10% FBS within a 12-well culture plate. A manual scratch was made in each well using a pipette tip. Cells were treated with 2.78 × 10^6^ om-hASCexo particles in triplicate. A Keyence BZX-800 microscope (Keyence, Itasca, IL, USA) was used to image the scratches. Images were taken in the same location at both timepoints. Keyence BZX-800 analyzer software v1.1.1.8 (Keyence, Itasca, IL, USA) was used to measure wound area.

### 2.7. Quantitative Real-Time PCR

RNA was isolated from samples using RNAzol (TelTest Inc., Friendswood, TX, USA) per manufacturer instructions. iScript (BioRad, Hercules, CA, USA, Cat #: 170-8891) was used to reverse transcribe RNA to cDNA. Maxima SYBR Green/Rox qPCR master mix (Applied Biosystems, Waltham, MA, USA, Cat #: A25742) was combined with 0.5 µL of cDNA for qPCR. The primers that were used are listed in Table 1. Real-time PCR was performed in triplicate. Amplification was completed with the ViiA 7 (Applied Biosystems, Waltham, MA, USA). Absolute quotient (AQ) or relative quotient (RQ) was calculated using optimized primer standard curves as indicated.

### 2.8. ProteinSimple Jess Automated Western Blot

To evaluate the presence of exosome markers Alix, CD9, CD63, and TSG101 in om-hASCexos, automated Western Blot was performed using ProteinSimple Jess system (ProteinSimple, Santa Clara, CA, USA) according to manufacturer instructions. The ProteinSimple 12–230 kDa Separation capillary cartridges (ProteinSimple, Santa Clara, CA, USA) were used for sample separation. In total, 1 mg/mL sample was loaded for each antibody. The antibodies listed in Table 2 were used at a 1:10 dilution. Automated Western Blot analysis was completed via Compass Software v7.0.0 (ProteinSimple, Santa Clara, CA, USA).

### 2.9. WST Assay

The cytotoxicity of cells exposed to H_2_O_2_ and then treated with om-hASCexos was evaluated via Abcam Cell proliferation WST-1 reagent (Cambridge, UK, Cat# ab155902) assay. Co-cultured HDF and HaCaT cells were plated on a 96-well plate at approximately 5 × 10^4^ cells/well and were left to incubate for 48 h to reach confluency. Cells were treated with 2 µL/well H_2_O_2_ (1:100 dilution) for 1 h. After H_2_O_2_ treatment, cell media was replaced, and cells were treated with 2.78 × 10^6^ om-hASCexo particles overnight. The following day, the media was changed, and 10 µL WST-1 reagents were added to each well and cells were incubated for 1.5 h. After incubation, the absorbance was measured at 480 nm. Treatments were performed in triplicate. Percent cytotoxicity was calculated as % Cytotoxicity = ((100 × (Control-Sample)))/Control.

### 2.10. Mitochondrial Stress Test

HDF cells were plated on a poly-D-lysine coated Seahorse XFp cell culture miniplate (Agilent Technologies, Santa Clara, CA, USA). Cells were plated at 4000 cells per well. HDF cells were then treated with 100 µM H_2_O_2_ for 30 min to induce oxidative stress. After 30 min, the media was changed to remove all H_2_O_2_, and cells were treated with 2.78 × 10^6^ om-hASCexo particles for 18 h. After 18 h of exosome treatment, the media was changed to the Seahorse XF Media. The plate was incubated at 37 °C in a non-CO_2_ incubator for 1 h. The Seahorse Extracellular Flux cartridge was prepped as instructed by the manufacturer. Calibration buffer was added to each well, and the plate was placed in the non-CO_2_ incubator at 37 °C for approximately 4 h. A total of 100 µM Oligomycin, 100 µM Fluoro-carbonyl cyanide phenylhydrazone (FCCP), and 50 µM Antimycin A/Rotenone were each added to the appropriate wells of the Flux cartridge. The Cell Mito Stress Test was then performed using the Seahorse XFp Analyzer. Oxygen consumption rates were measured at intervals of approximately 5–8 min. The measurements were normalized to protein concentration, which was quantified via Bradford assay. Data were analyzed using the Agilent Wave software v2.6.0.31. Measurement parameters (i.e., basal respiration, proton leak, non-mitochondrial oxygen consumption, etc.) were calculated as described by the manufacturer. Experiments were performed in duplicate and repeated twice. Average normalized values were calculated for analysis.

### 2.11. Chronic Inflammation

To induce chronic inflammation in cells, 5 ng/mL Lipopolysaccharide (LPS) was added to cell media for 6 h. After 6 h of inflammation, 2.78 × 10^6^ om-hASCexo particles were added to media for 4 days. Media was changed after 2 days. When media was changed, LPS and exosomes were added back into media. Cells were collected at the end of the 4-day treatment for qPCR analysis of inflammatory cytokines.

### 2.12. Animals

The James A. Haley Veteran’s Hospital and University of South Florida Institutional Animal Care and Use Committee (IACUC) approved all experimental procedures with animals consistent with the governing guidelines and recommendations of AWA and HREA. All experiments complied with the ARRIVE guidelines. All animals were raised and studied in pathogen-free environments housed in plastic, sawdust-covered cages with normal light–dark cycle and free access to chow and water.

### 2.13. Wounding and Exosome Treatment of Rats

Twelve-week-old male and female Fisher F344 rats were purchased from Jackson laboratories. Wounding of rats was completed as previously described [16]. Rats (*n* = 12, equal males and females) were wounded using a 6 mm biopsy punch in two standard locations on their back. Silicone rings were sutured around each wound to prevent skin constriction. Each wound was treated topically with either PBS vehicle control or 1.39 × 10^8^ om-hASCexo particles at day 0, then every other day until 7 days post-wounding. Wounds were dressed and then treated and re-dressed every other day. Photographs of wounds were taken at day 0 and repeated at each dressing change. Wounds were additionally measured using calipers. Rats were euthanized 7 days post-wounding, and the wounds and surrounding tissue were collected. Wound size was quantified by calculating the wound circumference (mm^2^). To determine the change in wound size over time, the percent wound closure was calculated each day. The In Vivo Imaging System (IVIS) was used to visualize the location of DIR-tagged exosomes every 2 days beginning at day 2 (48 h after first administration).

### 2.14. Immunohistocytology

Hematoxylin and Eosin (H&E; Abcam, Cambridge, UK, Cat #ab245880) and Masson’s Trichrome (Abcam, Cambridge, UK, Cat# ab150686) staining were performed per manufacturer instructions. Mean collagen area in the Trichrome images was determined using ImageJ v1.53. The density of microvessels was determined in H&E images by creating a grid on the 10× images and counting the number of vessels present in each quadrant.

To evaluate angiogenesis, we performed immunohistochemistry staining for VEGF. Tissue was blocked for 30 min using 1% BSA and 0.1% Triton X-100 in PBS. Wound tissue was incubated in the VEGF primary antibody (Santa Cruz Biotechnology, Dallas, TX, USA; Cat #sc-7269) at a 1:50 dilution for 24 h at 4 °C. After primary antibody incubation, tissue was incubated in the secondary antibody at a 1:1000 dilution for 2 h at room temperature prior to counter-staining with Hematoxylin for 30 s. Keyence analyzer software v1.1.1.8 was used to measure the total tissue area and the area of VEGF expression. Percent area of VEGF was quantified by dividing the VEGF area by the total tissue area.

### 2.15. RNA Sequencing of Wounds

RNA sequencing was completed as previously described [16]. RNA was isolated from the PBS- (Control) and om-hASCexo-treated wounds of two male and two female rats. Samples from each sex were pooled together for sequencing. RNA concentration and quality were measured using the Qubit (Thermo Fisher, Waltham, MA, USA) and Agilent Tape Station (RIN > 8.0; Agilent Technologies, Santa Clara, CA, USA). The TruSeq stranded mRNA Library Prep Kit was used according to the manufacturer’s instructions (Illumina, San Diego, CA, USA, Cat#: 20040532). The DNA libraries were checked for quality and concentration prior to loading samples into the Illumina NextSeq 500 with 75 bp pair-end reads with indices. The NextSeq System Suite (Illumina, San Diego, CA, USA) was utilized for real-time image analysis and base calling. All samples had a minimum of 40 million reads and sequences aligned to >80% of the reference genome. Trimmomatic was used to trim reads, and then a quality check was performed using FASTQC v0.12.1. Reads were mapped using HISAT2 v2.2.1 to rat genome GRCm39 (NCBI). Files were converted using SAMtools v 2023.09.0+463 and Feature-Counts v 2.20.0 were used to determine reads. RStudio (v 2023.09.0+463) was used for the analysis of differentially expressed genes (DEGs) with R package DESeq2 (v 1.42.1), and GSEA GO pathways were investigated using R package clusterProfiler (v 4.10.1). Established DEGs have a log2FC cutoff of −0.5 or 0.5 and an adjusted *p*-value less than 0.05.

### 2.16. Statistical Analysis

Experiments were repeated three times for biological replicates. Experimental samples were run in triplicate. Statistical analysis was performed as unpaired Student’s *t*-test, One-way, or Two-way ANOVA, as indicated using GraphPad Prism version 10.0.0 for Windows (GraphPad Software, Boston, MA, USA). * *p* < 0.05, ** *p* < 0.05, *** *p* < 0.001, and **** *p* < 0.0001 were used as significant measures.

## 3. Results

### 3.1. The Secretome from Om-hASCs Promotes Wound Healing In Vitro

**Figure 1:**
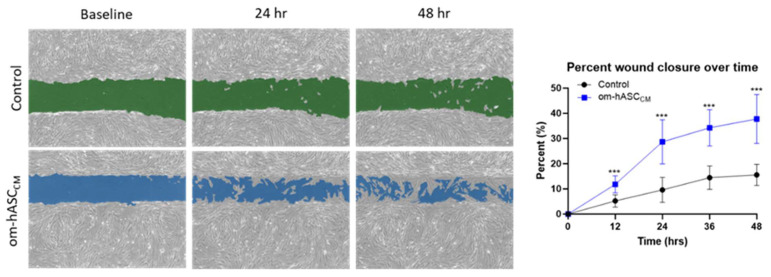
A scratch assay was performed on HDF cells (*n* = 3). Cells were treated with CM from om-hASCexo to evaluate the effectiveness of the secretome for treating wounds. The gap between cells was measured using the Keyence analyzer software v1.1.1.8. Statistical analysis was performed pairwise via Student’s unpaired *t*-test. Significant *p*-values (<0.05) are indicated. *** *p* < 0.001.

Prior clinical research has described the regenerative and immunological properties of omental adipose tissue in the repair of internal injuries. To understand the underlying mechanisms, we evaluated the secretome collected as conditioned media (CM) from human omental adipose stem cells. The om-hASC CM was used in the in vitro scratch assay to evaluate its ability to close cellular gaps independent of the om-hASCs. The results (Figure 1) demonstrate that om-hASC-derived CM significantly accelerated the closure of gaps by over 100% compared to control cells.

### 3.2. Characterization of Om-hASCexos

**Figure 2:**
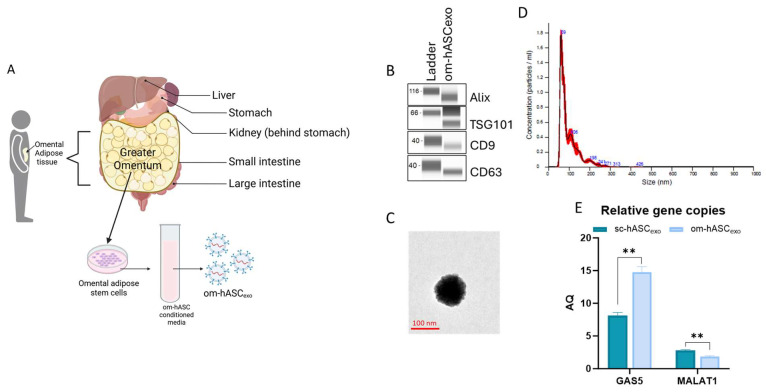
(**A**) Schematic depicting the omentum adipose depot and isolation of om-hASCexos, created in BioRender. Patel, N. (2025) https://BioRender.com/y0k3g3h, accessed on 18 August 2025. Copyright 2025, Patel, N.A. Publication license can be found in Supplementary Materials S1. (**B**) Automated Western Blot was performed to verify the presence of exosome surface proteins including CD9, CD63, TSG101, and Alix on om-hASCexos. Experiments repeated thrice and representative images are shown. (**C**) TEM image of om-hASCexo. (**D**) om-hASCexo diameter was evaluated via NanoSight v3.2.01. (**E**) Levels of lncRNAs *GAS5* and *MALAT1* determined through dPCR in copies per μg of exosome in om-hASCexos compared to sc-hASCexos. Statistical analysis was performed pairwise via Student’s unpaired *t*-test. Significant *p*-values (<0.05) are indicated. ** *p* < 0.005.

Small extracellular vesicles (sEVs) were isolated from the CM of om-hASCs (Figure 2A). We then determined the properties of the small extracellular vesicles (sEVs) derived from the CM of om-hASCs to validate that the particles are exosomes. The presence of exosome surface markers CD9 and CD63 and the endosomal sorting complex required for transport (ESCRT) proteins TSG101 and Apoptosis-linked gene 2-interacting protein X (Alix) were evaluated via Automated Western Blotting (JESS, ProteinSimple, Santa Clara, CA, USA). The results show the presence of these markers, thus confirming that the sEVs isolated from CM are exosomes (Figure 2B). Full, unaltered blots can be found in Supplementary Materials S4.

The morphology of the om-hASCexos was visualized using Transmission Electron Microscopy (TEM) (Figure 2C). The vesicle diameter and concentration were evaluated via NanoSight. The average size of om-hASCexos is approximately 95 nm (range is 59–106 nm; Figure 2C). Exosomes measure between ~40 and 160 nm, further confirming that the EVs are exosomes. NanoSight Analysis revealed that the total concentration of exosomes was approximately 6.96 × 10^8^ exo/mL (Figure 2D).

We previously demonstrated [8] the differences in the lncRNA content of exosomes derived from omental hASC versus subcutaneous hASC depots. We have previously published that long noncoding RNA (lncRNA) *MALAT1* and *GAS5* were highly enriched in exosomes derived from the subcutaneous (sc) depot. Here, digital PCR (dPCR) was used to evaluate the relative gene copies of *GAS5* and *MALAT1* in om-hASCexos and compared the amount present in sc-hASCexos. The results showed that om-hASCexos contain approximately 50% more *GAS5* copies/μg of exosome than sc-hASCexos. *MALAT1* was packaged at a lower level compared to *GAS5* in both om- and sc-hASCexos (Figure 2E). Overall, this variation in enrichment of the lncRNAs *GAS5* and *MALAT1* in om-hASCexos and sc-hASCexos demonstrates that exosomes originating from different adipose depots carry differing levels of their genetic cargo.

### 3.3. Proteomic Analysis of Om-hASCexos

**Figure 3:**
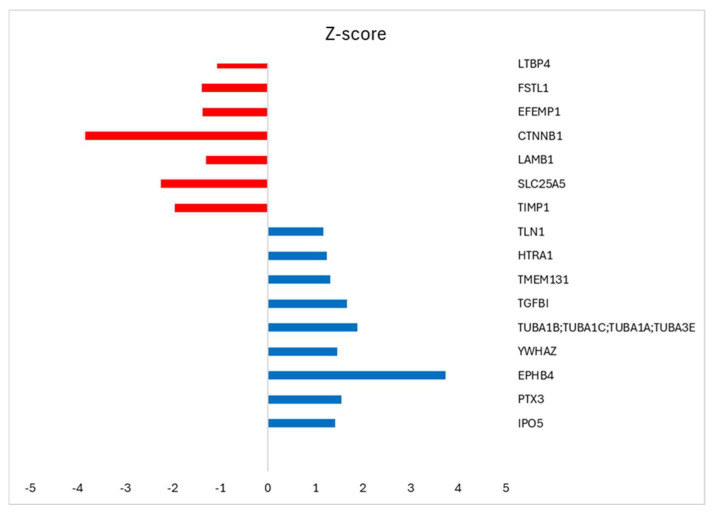
Perseus software v2.1.5 was used to analyze the proteomic results from subcutaneous-derived hASCexos and the om-hASCexos. The log LFQ intensity data of the commonly expressed proteins within both hASCexo and om-hASCexo preparations was used to determine the ratio hASCexo/om-hASCexo. GraphPad was used for statistical analysis of data with Welch’s *t*-test, and z-score was calculated. *p* < 0.5 was considered significant. Results identify the top highly expressed genes in hASCexos, with z-score > 1 (blue bar), and the highly expressed genes in om-hASCexos, with z-score < 1 (red bar).

The protein composition of om-hASCexos was evaluated using mass spectrometry. The enrichment was determined using the Log label-free quantification (LFQ) intensity as calculated by Perseus software v2.1.5. The highly enriched proteins are listed in Table 3. Additionally, we compared the protein composition between the exosomes from the subcutaneous adipose stem cells (sc-hASCexos) and om-hASCexos to evaluate cargo differences. The mass spectrometry results were analyzed, and the ratio of exosomal proteins in sc-hASCexos to om-hASCexos was calculated. The top proteins enriched in sc-hASCexos (blue) and in om-hASCexos (red) were identified by the z-score (Figure 3).

### 3.4. Om-hASCexos Significantly Improve Cell Migration In Vitro

**Figure 4:**
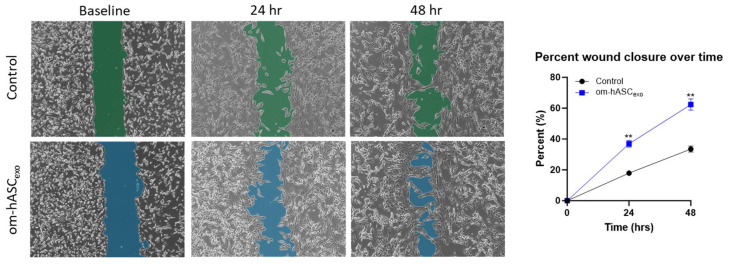
A scratch assay was performed on HDF cells (*n* = 3). Cells were treated with exosomes derived from om-hASC. The gap between cells was measured using the Keyence analyzer software v1.1.1.8. Statistical analysis was performed pairwise via Student’s unpaired *t*-test. Significant *p*-values (<0.05) are indicated. ** *p* < 0.005.

We undertook a series of assays mimicking physical and biological injuries in vitro to establish the repairing and regenerative ability of om-hASCexos. Since we were using a dermal injury model for the in vivo studies, we used dermal fibroblasts (HDFs) to align our in vitro experimental results to the in vivo studies. To evaluate the effect on cell migration, a scratch assay was performed on HDF cells. A gap was introduced using a pipette tip. After 24 h, the cells treated with om-hASCexos had a significantly greater gap closure percentage than untreated cells. This indicates that om-hASCexos accelerate cell migration (Figure 4).

### 3.5. Om-hASCexo Treatment Attenuates Oxidative Stress In Vitro

**Figure 5:**
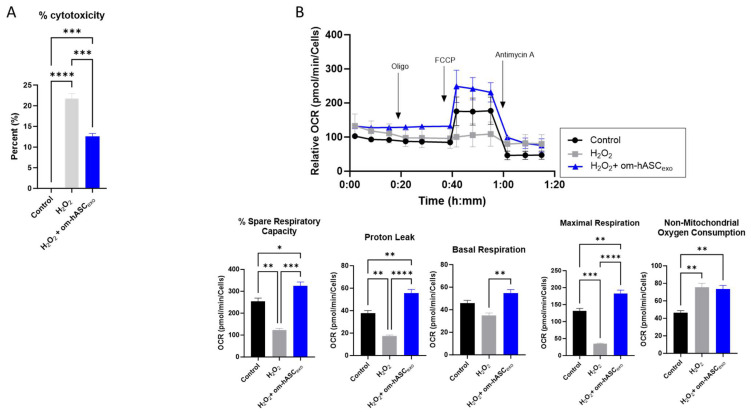
(**A**) HDF cells were treated with 1 μL/mL (1:1000) H_2_O_2_ for 1 h, then treated with exosomes overnight. Abcam cell proliferation WST-1 reagent assay was used to evaluate the cytotoxicity of cells under H_2_O_2_ insult and with exosome treatment (*n* = 3). (**B**) HDF cells were seeded in a Seahorse XFp miniplate and treated as described above. A Mito Stress Test Assay was performed according to the manufacturer’s instructions. The results were normalized to the protein content of each well. Seahorse Wave software v2.6.0.31 was used for analysis (*n* = 3). Statistical analysis was performed via One-way ANOVA. Significant *p*-values (<0.05) are indicated on each graph. * *p* < 0.05; ** *p* < 0.005; *** *p* < 0.001; **** *p* < 0.0001.

To evaluate the effect of om-hASCexos on cytotoxicity, HDF cells were treated with 2 µL/mL H_2_O_2_ (1:1000 dilution) for 1 h. The cell medium was removed, and a fresh medium was added along with a 2 μg om-hASCexo treatment. The WST-1 assay was performed to evaluate the om-hASCexos’ ability to rescue cells from the cytotoxicity that results from oxidative stress. The addition of H_2_O_2_ to the cells resulted in a 21% cytotoxicity (Figure 5A). However, the cytotoxicity was rescued by om-hASCexo treatment post-H_2_O_2_ to 12%. This indicates that om-hASCexo treatment rescues cells from oxidative stress-induced toxicity.

To gain further insight into the effects of om-hASCexos on mitochondria during oxidative stress, a Seahorse mitochondrial stress test was performed (Figure 5B). HDF cells were plated in the Seahorse cell culture plate; the cells were treated with 2 µL 1:100 diluted H_2_O_2_ per well for 30 min, then the cell media was changed and a 2.78 × 10^6^ om-hASCexo particle treatment was added overnight to each well. Compared to untreated control cells, the cells treated with H_2_O_2_ had a significantly reduced percent spare respiratory capacity, maximal respiration, and proton leak. Basal respiration was not significantly different. However, when H_2_O_2_-treated cells where additionally treated with om-hASCexos, the percent spare respiratory capacity, basal respiration, maximal respiration, and proton leak all increased to levels equal to or greater than that of the control cells, indicating that om-hASCexos rescued these cells from oxidative stress. There was no significant change in the non-mitochondrial oxygen consumption when comparing H_2_O_2_ and H_2_O_2_ + om-hASCexo treatments, though both were significantly greater than the control.

### 3.6. Om-hASCexo Treatment Attenuates LPS-Induced Inflammation In Vitro

**Figure 6:**
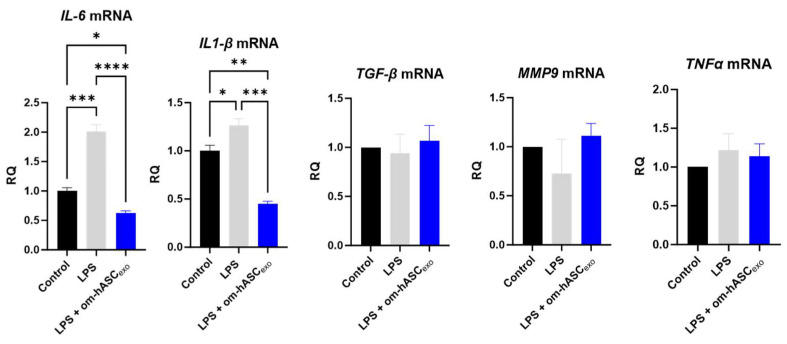
HDF cells were treated with 5 ng/mL LPS for 6 h, then exosomes were added to media for 4 days. Media was changed every 48 h. At each media change, LPS and om-hASCexos were added to each well (*n* = 3). Statistical analysis was performed via One-way ANOVA. Significant *p*-values (<0.05) are indicated on each graph. * *p* < 0.05; ** *p* < 0.005; *** *p* < 0.001; **** *p* < 0.0001.

The effect of om-hASCexos on inflammation was evaluated. To induce chronic inflammation, 5 ng/mL LPS was added to the cell media for 6 h prior to the om-hASCexo treatment. After 4 days of LPS and om-hASCexo treatment, pro-inflammatory cytokines *IL6* and *IL1-β* were significantly increased in the LPS treated cells, and the treatment with om-hASCexos reduced (Figure 6) the levels of the inflammatory genes, indicating that om-hASCexos decreased inflammatory pathways. *MMP9*, *TGFβ*, and *TNFα* did not increase with LPS treatment, probably due to the short duration of the LPS treatment.

### 3.7. Om-hASCexo Treatment Improved Wound Healing Rate in an In Vivo Rat Model

**Figure 7:**
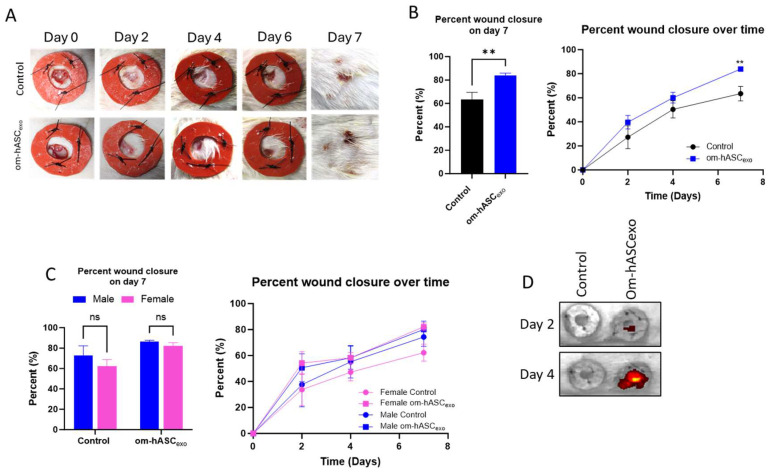
Male and female rats were used for an in vivo model of wound healing with om-hASCexo treatment. Two 6 mm wounds were created on the back of each rat using a biopsy punch. The wounds were treated on day 0, then every 2 days for 7 days. On day 7, the rats were euthanized, and wound tissue was collected for analysis. (**A**) Photos were taken and (**B**) wounds were measured with calipers every 2 days to monitor wound healing progress. The percent wound closure was calculated, and analysis was conducted for day 7 post-wounding and over the 7 days for all rats (*n* = 9). (**C**) Analysis was completed to compare wound healing on day 7 and over time in male and female rats (*n* = 4–6). (**D**) In Vivo Imaging System was used to visualize the location of DIR-marked exosomes for days 2 and 4. Statistical analysis was performed pairwise via Student’s unpaired *t*-test, or One-way or Two-way ANOVA where appropriate. ns = not significant. Significant *p*-values (<0.05) are indicated on each graph. ** *p* < 0.05.

Next, the efficacy of om-hASCexos was tested in vivo using a rat dermal wound model. Two 6 mm wounds were created on the back of each rat using a biopsy punch. Wounds were treated every 2 days with exosomes. They were measured using calipers to monitor the healing rate. Overall, the wounds treated with om-hASCexos experienced quicker healing, with a significantly greater wound closure percentage at day 7 compared to the control wounds (Figure 7A,B). At day 2 post-wounding, the wound closure in male rats was greater than that of female rats. Additionally, on day 2, males appear to have a greater response to om-hASCexos than females. However, by day 4, the wound closure percents for male and female rats were similar. On day 7, there was no significant difference between the rate of healing for males and females between each treatment group (Figure 7C). To evaluate the movement of topically applied exosomes, we applied DIR-tagged exosomes and visualized the fluorescence every 2 days prior to the application of fresh exosomes. The exosomes remain in the area of the wound, with little dispersion to other body organs (Figure 7D). This indicates that om-hASCexos are predominantly retained at injury sites.

### 3.8. Om-hASCexo Treatment Increased Angiogenesis in Wounds

**Figure 8:**
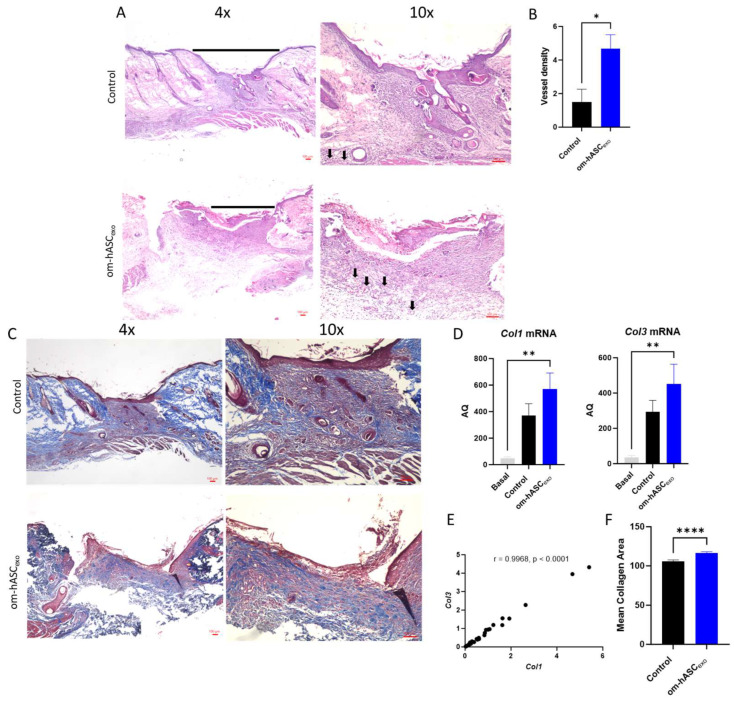

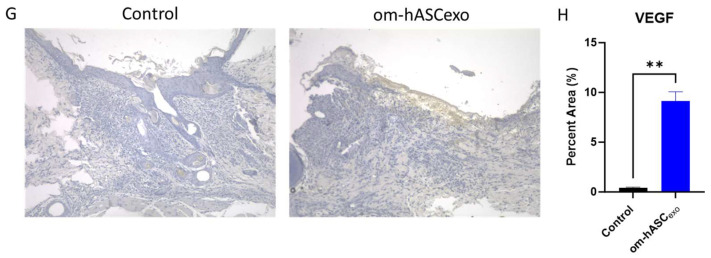
(**A**) Hematoxylin and Eosin (H&E) staining was completed to evaluate the morphological differences and presence of microvessels in control wounds and those treated with om-hASCexos. Black horizontal lines indicate the wound bed, and black arrows indicate microvessels. The red scale bar in the bottom right of the images represents 100 µm. (**B**) Microvessel density was quantified by creating a 3 × 4 grid over the 10× wound image and counting the number of microvessels in each quadrant. The average number of microvessels was calculated per slide using the Keyence Software v1.1.1.8. (**C**) Masson Trichrome staining was completed to visualize the variation in collagen deposition between groups. The red scale bar in the bottom right of the images represents 100 µm. (**D**) SYBR Green RT qPCR analysis of *Col1* and *Col3* was completed for collagen mRNA level quantification. Relative Quantification (RQ) was determined using a control sample for reference. (**E**) The correlation between *Col1* and *Col3* was calculated. (**F**) Mean collagen area was quantified in Masson’s Trichrome staining images using ImageJ v1.53 (*n* = 3). (**G**) VEGF immunohistochemistry staining. Brown areas indicate the VEGF; blue areas are the nuclei. (**H**) Percent area of VEGF expression (*n* = 3). Statistical analysis was performed pairwise via Student’s *t*-test or One-way ANOVA, where appropriate. Significant *p*-values (<0.05) are indicated on each graph. * *p* < 0.05, ** *p* < 0.005; **** *p* < 0.0001.

Wounds were collected on day 7. H&E staining was conducted to evaluate the cellular composition and morphology (Figure 8A). The H&E staining indicated less cellular recruitment in the wound that was untreated (control) compared to the wound treated with om-hASCexos. A further analysis of the images revealed the generation of microvessels in the om-hASCexo-treated wounds, demonstrating increased angiogenesis (Figure 8B).

To evaluate the wound bed for collagen deposition, Masson Trichrome staining was performed (Figure 8C). The results showed an increase in collagen fibers (blue stain) in the om-hASCexo-treated wounds. To verify, *Col1* and *Col3* levels in the wound were quantified via qPCR. The qPCR results revealed that the wounding of the skin significantly increased both *Col1* and *Col3* from basal (Day 0) conditions (Figure 8D). *Col1* and *Col3* levels were greater in wounds treated with om-hASCexos compared to the control wounds, indicating a quicker wound healing. Furthermore, the correlation between *Col1* and *Col3* was evaluated, and a strong positive correlation was found (Figure 8E). The collogen composition revealed in the Masson’s Trichrome stain was quantified using Image J v1.53. The quantification of stained collagen revealed a similar trend as *Col1* and *Col3* mRNA, with an increase in collagen in the samples treated with om-hASCexos (Figure 8F).

The wounds were also analyzed for VEGF deposition. Tissue was incubated with a VEGF IHC antibody. The tissue was visualized and analyzed using a Keyence BZX-800 microscope and software v1.1.1.8. The analysis revealed that there was significantly greater VEGF expression in the wounds of rats treated with om-hASCexos, indicating a greater prevalence of angiogenesis (Figure 8G,H).

### 3.9. Om-hASCexo Treatment Acts upon Pathways Associated with Immune Response, Lipid and Fatty Acid Metabolism, Apoptosis, and Cell Differentiation

**Figure 9:**
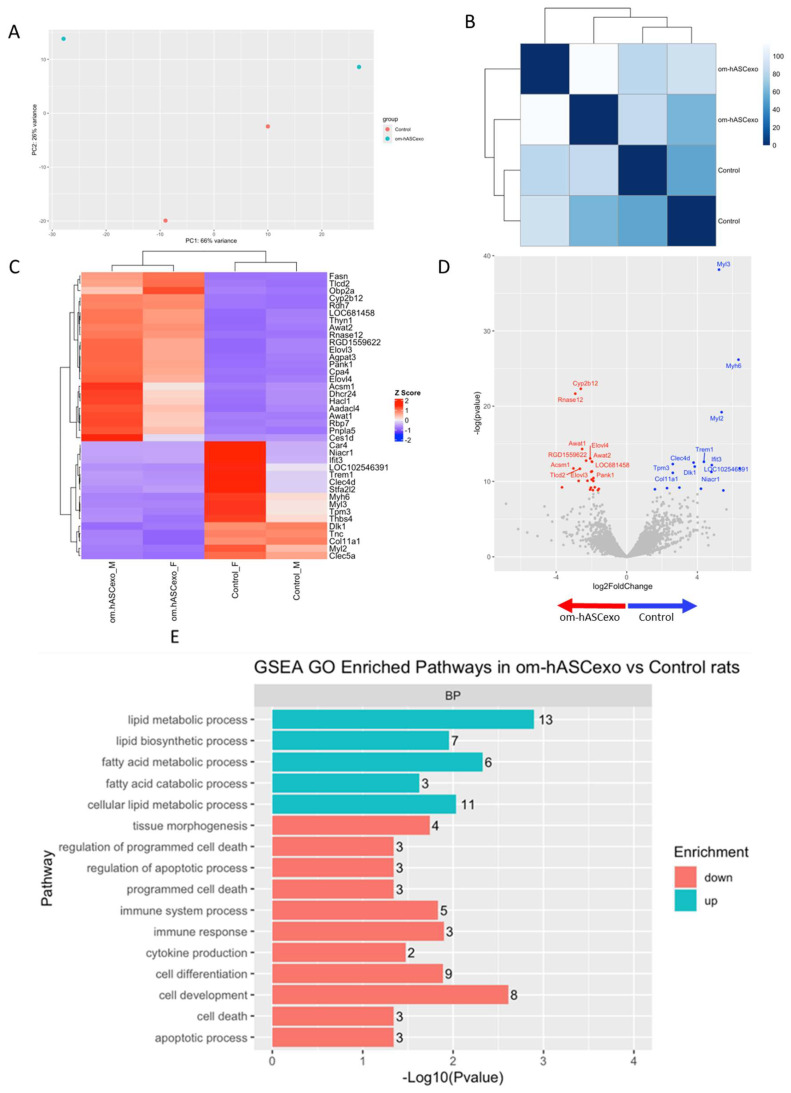

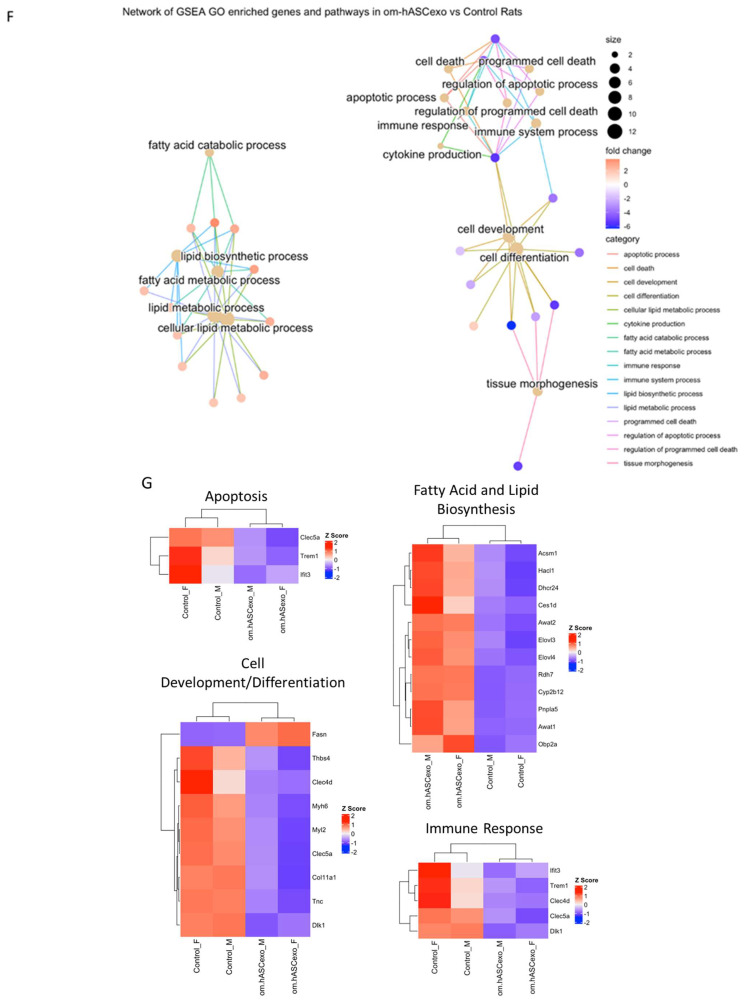

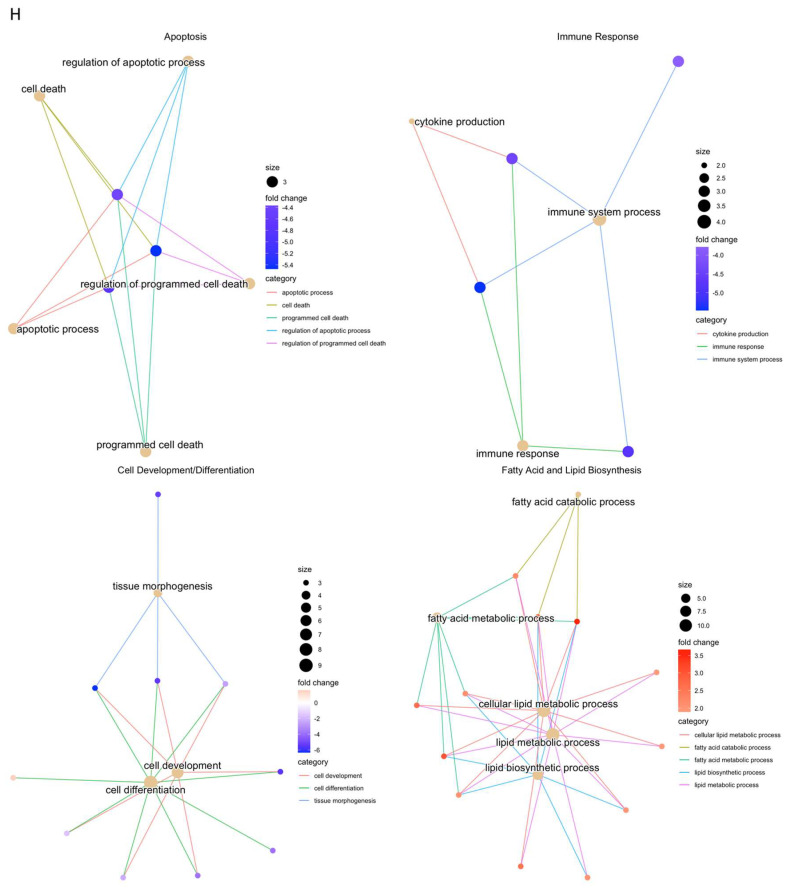
RNAseq analysis was performed using wound samples from the in vivo rat model to compare om-hASCexo-treated wounds vs. control wounds (*n* = 2). (**A**) To evaluate the variance and similarity between treatment groups and samples within a group, a PCA plot and (**B**) heatmap of sample similarity were created. (**C**) A differentially expressed gene (DEG) heatmap and (**D**) volcano plot were created to evaluate significant DEGs between om-hASCexo-treated wounds and control wounds. Genes labeled in blue are downregulated and those labeled in red are upregulated in om-hASCexo wounds compared to control wounds. (**E**) To evaluate the enriched pathways in om-hASCexo wounds compared to control wounds, a Gene Set Enrichment Analysis (GSEA) Gene Ontology (GO) was conducted on Biological Processes (BP) pathways. The GSEA GO-enriched pathways of interest are depicted. Red bars indicate pathways that are downregulated and blue bars indicate pathways that are upregulated in wounds treated with om-hASCexos compared to control wounds. The numbers at the ends of bars indicate the number of significant genes present in that pathway. Bar length is determined by the −Log10(*p* value) to show the significance of each pathway. (**F**) To visualize the relationship between each pathway, a network plot of all GSEA GO pathways of interest was created. The tan dots indicate each pathway of interest while the connected red and blue dots indicate the genes included in those pathways. The size of each tan (pathway) dot indicates the number of significant genes enriched in these samples included in these pathways. The color of the red/blue (gene) dots indicates the fold change, or whether the gene is up- or downregulated in that pathway. (**G**) From the 16 enriched pathways of interest, 4 overall pathway groups were created: Apoptosis, Fatty Acid and Lipid Biosynthesis, Cell Development/Differentiation, and Immune Response. Pathways were grouped into these categories, and heatmaps of gene expression were created for each pathway group to evaluate which significant genes are enriched in the related pathways. Positive Z values (red) indicate gene upregulation while negative Z values (blue) indicate gene downregulation. (**H**) Network plot was created for each pathway group to visualize how the genes enriched in these pathways are expressed. The tan dots indicate each pathway of interest, while the connected red and blue dots indicate the genes included in those pathways. The size of each tan (pathway) dot indicates the number of significant genes enriched in these samples included in these pathways. The color of the red/blue (gene) dots indicates the fold change, or whether the gene is up- or downregulated in that pathway.

RNAseq was performed on the wounds collected on day 7 to evaluate the differentially expressed genes and pathways in wounds treated with om-hASCexos compared to control wounds. The Principal Components Analysis (PCA) and distance matrix analysis indicate clustering between the sample replicates and an adequate distance between the treatment groups (Figure 9A,B). Thirty-eight significant differentially expressed genes (DEGs) were identified, with fifteen genes downregulated and twenty-three genes upregulated in om-hASCexo-treated wounds compared to control wounds (Figure 9C). Genes were determined to be DEGs if they had a log2FC cutoff of −0.5 or 0.5 and an adjusted *p*-value less than 0.05. A negative log2FC value indicates that the gene is upregulated in om-hASCexo-treated wounds compared to control wounds. A positive log2FC value indicates that the gene is upregulated in the control wounds compared to wounds treated with om-hASCexos. The top 5 DEGs with a log2FC cutoff of −0.5 or 0.5 and an adjusted *p*-value less than 0.05 include *Cyp2b12*, *Rnase12* (both upregulated), *Myl3*, *Myh6*, and *Myl2* (downregulated) (Figure 9D).

To identify the functionality of om-hASCexos in wound healing, Gene Set Enrichment Analysis (GSEA) in Biological Processes (BP) from Gene Ontology (GO) pathways was performed. These pathways were divided into four main pathway categories, as described in Table 4. GSEA GO analysis indicated that the pathways involved in cell development and differentiation, apoptosis, and immune response were suppressed in om-hASCexo-treated wounds compared to the control wounds. On the other hand, the pathways involved in fatty acid and lipid metabolic and biosynthetic processes were shown to be activated in wounds treated with om-hASCexos (Figure 9E). A network analysis of these pathways and their related genes was conducted to visualize how these pathways are connected (Figure 9F). Heatmaps of the enriched genes in each of these pathway categories were created to better visualize how om-hASCexo treatment affects the genes involved in these pathways to promote wound healing (Figure 9G), and individual network analyses of these grouped pathways were performed (Figure 9H). Raw RNAseq count data can be found in Supplementary Materials S3.

### 3.10. Om-hASCexo Treatment Affected Expression of Pro-Inflammatory and Anti-Apoptotic Factors 7 Days Post-Wounding In Vivo

**Figure 10:**
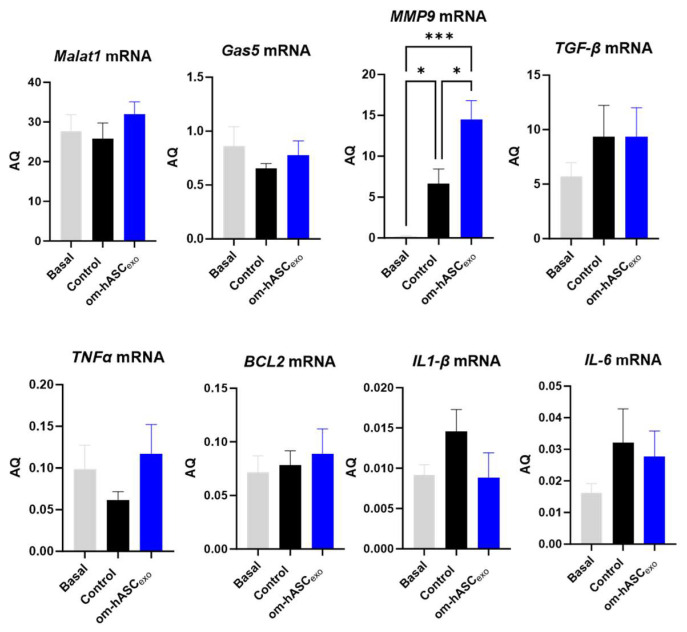
Rat wound samples were processed for SYBR Green RT qPCR of *Malat1*, *Gas5*, *MMP9*, *TGF-β*, *TNFα*, *BCL2*, *IL1-β*, and *IL-6* (*n* = 5). AQ was calculated using a standard curve. Statistical analysis was performed pairwise via One-way ANOVA. Significant *p*-values (<0.05) are indicated on each graph. *** *p* < 0.001; * *p* < 0.05.

qPCR was performed on day 7 samples for endogenous levels of lncRNAs *Malat1* and *Gas5*, pro-inflammatory factors *MMP9*, *TGF-β*, *TNFα*, *IL1-β*, and *IL-6*, and anti-apoptotic factor *Bcl2* (Figure 10). The results show that wounding slightly decreased the endogenous levels of *Gas5* and *Malat1* compared to the basal (non-wounded) samples. The treatment with om-hASCexos increased the *Gas5* and *Malat1* levels to the basal amounts. *Mmp9* levels were significantly increased in wounds compared to healthy tissue and increased further under treatment. The levels of *TGFβ* were increased in the wounds and did not change significantly with om-hASCexo treatment at day 7. *TNFα* levels decreased in the wound bed at day 7 but increased with the om-hASCexo treatment. However, *IL-1β* and *IL-6* levels increased 7 days post-wounding and were slightly decreased with the om-hASCexo treatment. *Bcl2* levels were not altered after 7 days of wounding nor when wounds were treated with om-hASCexos.

## 4. Discussion

Adipose depots have distinct functions associated with their location in the human body. The subcutaneous depot, due to its location directly beneath the skin, has been extensively studied. Less is known about the mechanisms of action of the internal adipose depots, such as the omentum and its ability to regulate injury to internal organs. While the omental adipose depot is established as a crucial source of immune response and regulation of inflammation in the clinic, the underlying molecular mechanism is not well known. In this study, we postulated that the omentum’s ability to repair and rejuvenate internal trauma is via its secretome. Hence, we evaluated the exosomes and nanovesicles secreted by the omental adipose stem cells, and their role in protecting and repairing the recipient cells post-trauma. Recent studies on exosomes originating from obese adipose tissue have shown that these exosomes promote the progression of multiple cancers, metabolic disorders, insulin resistance, and atherosclerosis [17,18]. Here, our study using lean, normal omental-derived exosomes showed the difference in cargo which may affect hyperglycemia, atherosclerosis, and other conditions. In the future, we can address the mechanisms at play in disease progression.

**Figure 11:**
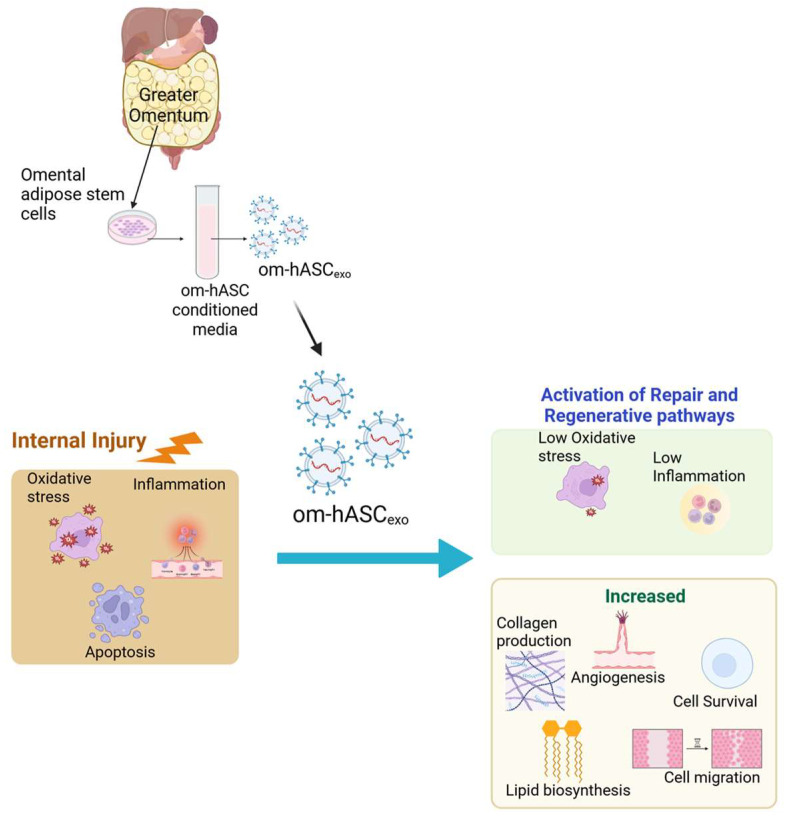
Schematic depicting that om-hASCexos improve healing post-injury by decreasing oxidative stress and inflammation and increasing angiogenesis and cell survival; created in BioRender (Patel, N. (2025) https://BioRender.com/hgprsu7), accessed on 18 August 2025. Copyright 2025, Patel, N.A. Publication license can be found in Supplementary Materials S2.

Controlling inflammation in internal injuries is critical for the healing process [19,20,21]. In addition to uncontrolled inflammation, chronic wounds are also characterized by impaired angiogenesis or re-epithelialization, the dysregulation of cytokines or growth factors, and/or increased protease activity [22]. When inflammation becomes uncontrolled, it can delay the healing process due to a breakdown of the extracellular matrix of tissues [23]. Ultimately, this results in a vicious cycle of recalcitrant wounds which lead to chronic damage to tissues and organs. In this study, we demonstrate that om-hASCexos decrease inflammation and oxidative stress for in vitro and in vivo wound models (Schematic in Figure 11).

Another factor that can alter normal healing is oxidative stress, as excessive amounts can further exacerbate inflammation [24]. Our results demonstrated that om-hASCexos decreased oxidative stress. Oxidative stress occurs when there is an over-abundance of reactive oxygen species (ROS) and a depletion of antioxidants [25]. Oxidative stress can result in issues including cell membrane damage, lipid peroxidation, and necrotic cell death [26]. Arya et al. (2011) found that, in patients with uncontrolled diabetes, which is known to cause increased oxidative stress, apoptosis activator Caspase-3 levels were increased, and anti-apoptotic factor Bcl-2 was suppressed compared to healthy, non-diabetic patients [26]. Studies, such as that of Zhou et al. (2021), have demonstrated how oxidative stress decreases cell viability and migration [27]. Furthermore, Li et al. (2019) previously found that, in rats, by suppressing factors which protect against both oxidative stress and inflammation, dermal wounds healed significantly slower [28]. By controlling inflammation and oxidative stress, healing can be accelerated, preventing ongoing complications from internal injuries.

Angiogenesis is an important function in wound healing, as new blood vessels carry oxygen and nutrients to the wound. Delayed angiogenesis, as seen in diabetic patients, in turn results in delayed healing. In this study, we found evidence of increased angiogenesis occurring in wounds treated with om-hASCexos. In the tissue stained via H&E, there are a greater number of vessels present around the wound bed in om-hASCexo-treated wounds compared to the control wounds, demonstrating an increased angiogenesis. Vascular endothelial growth factor (VEGF) is critical to angiogenesis in wound healing. VEGF levels increase immediately after wounding, peak at 3–5 days, and then decrease to normal levels [29]. Here, we performed IHC for VEGF. We found that there were significantly greater levels of VEGF present in wounds treated with om-hASCexos compared to the control wounds, indicating a greater angiogenesis. VEGF is also expressed in keratinocytes in the epidermis [30].

Collagen type I (Col1) and collagen type III (Col3) are the major types of collagen found in the extracellular matrix (ECM). While Col1 functions in providing tensile strength and structural support to the ECM, Col3 allows for tissue elasticity [31]. Col1 and Col3 are known to be closely associated during wound healing, with the Col1/Col3 ratio changing throughout the healing process. In the beginning phases of wound healing, Col3 is more prominent, and by the end of the healing process Col1 has become dominant [32]. Here, we confirmed a strong positive correlation between *Col1* and *Col3* in the wound bed at 7 days post-wounding, including when treated with om-hASCexos (Figure 8E). This indicates that the treatment of wounds with om-hASCexos does not alter this relationship between *Col1* and *Col3* by day 7 of healing.

Previous studies have indicated lncRNAs, metastasis-associated lung adenocarcinoma transcript 1 (*MALAT1*), and growth-arrest specific-5 (*GAS5*) as highly enriched in subcutaneous hASCexos (sc-hASCexos). Furthermore, previous data have shown that *MALAT1* and *GAS5* are beneficial to healing [20,33,34]. Our results demonstrate that *GAS5* is highly enriched in exosomes derived from the omental adipose depot. *MALAT1*, on the other hand, is slightly less enriched in om-hASCexos compared to sc-hASCexos, though is still a highly enriched lncRNA within exosomes. In vitro data indicates that *MALAT1* increases cell proliferation and migration and inhibits apoptosis [33,35], while *GAS5* reduces inflammation via the inhibition of pro-inflammatory molecules including IL6 and IL1-β [20]. Additionally, *GAS5* levels are depleted in diabetic patients [36], resulting in an inhibition of glucose uptake and insulin signaling [30], both of which are critical to normal healing. Both *GAS5* and *MALAT1* have been extensively studied in the field of cancer research. In multiple cancers, *GAS5* is a known tumor suppressor in which high levels are associated with increased apoptosis and decreased cell proliferation [37]. However, *GAS5* appears to function differently in stem cells. Xu et al. (2016) found that an overexpression of *GAS5* in human embryonic stem cells promoted self-renewal [38]. *MALAT1* has proven to be important for the promotion of the metastasis of various cancers, such as colorectal cancer [39] and pancreatic cancer [40], whereas it has been shown to suppress metastasis in breast cancer [41] and glioma [42]. Li et al. (2016) found that, in pancreatic cancer, *MALAT1* activates functions associated with metastasis, such as autophagy, migration, apoptosis, and cell invasion [40]. Interestingly, these functions are also beneficial to the healing of both dermal and internal injuries. Overall, it appears in these data that, by treating cells with om-hASCexos highly enriched in *GAS5* and *MALAT1*, inflammation is suppressed and proliferation and migration are promoted.

In addition to evaluating om-hASCexo cargo for lncRNAs *GAS5* and *MALAT1*, we also employed proteomic techniques to elucidate the protein cargo. The top abundant proteins, listed in Table 3, are shown to be present in exosomes from mesenchymal stem cells (ExoCarta: A web-based compendium of exosomal cargo) [43]. Of interest were the proteins that were contained in higher levels in om-hASCexos compared to sc-hASCexos. The Latent Transforming Growth Factor Beta Binding Protein 4 (LTBP4) binds to TGFβ, a key protein in wound healing, to maintain it in the latent stage until insertion into extracellular matrix [44,45]. It also plays an important role in elastic fibers [46,47], thus promoting repair and regeneration. Beta-catenin (CTTNB1) was significantly higher in om-hASCs, and points to the promotion of cell adhesion and communication between cells, and the activation of lipid metabolism in adipose tissue [48]. Solute carrier family 25 member 5 (SLC25A5) is a mitochondrial protein involved in the translocation of ATP and ADP, and is also found in higher levels in om-hASCexos compared to sc-hASCexos, implicating an increased energy metabolism. TIMP1 inhibits the activity of matrix metalloproteinases, thereby maintaining a balance to protect the extracellular matrix. Bao et al. demonstrated that TIMP1 is required for myometrial contractions during labor [49]. The abundance of these proteins in om-hASCexo cargo provides some insight into how om-hASCs function to promote internal healing as well as the regulation of metabolic pathways.

In our RNAseq analysis of samples post-application of om-hASCexos, we found that several biological pathways involved in various metabolic and biosynthetic processes were upregulated in om-hASCexo wounds compared to the control wounds. These pathways include the fatty acid and lipid biosynthetic and metabolic pathways. Lipids such as cholesterol and fatty acids are vital to the skin’s outermost layer, the stratum corneum, as they prevent desiccation [50]. During wound healing, lipids are known to regulate processes such as inflammation, angiogenesis, proliferation, migration, and tissue repair [51]. Our results here indicate that, 7 days after wounding, om-hASCexo treatment functions in increasing the activation of fatty acid and lipid biosynthesis and metabolism to promote faster healing than in untreated wounds.

Other important pathways involve the immune response pathway. Many studies have indicated that, near the end of the inflammation phase, the apoptosis of immune cells is necessary to move through the healing process [26,52]. The suppression of apoptosis by om-hASCexos would indicate a further progression through the wound healing process. These groups of pathways were analyzed for any repeated or prominent significant genes. One such gene that was present between apoptosis, cell differentiation, and immune response pathways was *Clec5a*. Human C-type lectin domain family 5 member A (Clec5a) is highly expressed in myeloid cells [53] and is known to be involved in processes including cell adhesion and cell growth [54], both of which are important for the healing of wounds. Further, it has been demonstrated that, in certain types of cancers, Clec5a promotes tumorigenesis through the activation of the PI3K/AKT pathway [54,55]. The results presented here show that, on day 7 post-wounding, om-hASCexo treatment resulted in a downregulation of *Clec5a*, indicating that, at this point in the healing process, cell adhesion and growth have begun to slow. Another significant gene that is repeated in both apoptosis and immune response related pathways is Triggering receptor expressed on myeloid cells-1 (*Trem1*). Trem1 is expressed in most innate immune cells [56] and is known to trigger the production of pre-inflammatory cytokines [57]. Previously, it was believed that Trem1 primarily played a role in infectious diseases, but recently it has been determined that soluble Trem1 is a mediator of several inflammatory diseases, including colitis, rheumatoid arthritis, psoriasis, and sepsis [56,58]. The RNAseq results here show that om-hASCexo treatment results in the downregulation of *Trem1* on day 7, indicating a decrease in inflammatory cytokine production. Both Clec5a and Trem1 appear to hold multiple functions within wound healing. Further explorations into the mechanisms of how om-hASCexos affect Clec5a and Trem1 are currently in progress.

Previous studies on exosomes have demonstrated that intravenous administration via the tail vein has indicated that florescence-labeled exosomes are cleared from the blood within 4 h and are then detected in the liver, spleen, lungs, and gastrointestinal tract [59,60]. It has been proposed that a topical application to the skin, eyes, or mucosal surfaces may have a shorter half-life due to the exposure of exosomes to external factors or bodily fluids such as tears or sweat [61]. However, there have been many studies of the benefits of topical exosome treatments in dermatological cases [62], indicating that their half-life is adequate for topical treatments. Our data (Figure 7) show that exosomes are retained at the site of injury.

A rat model of dermal healing was used in this study, as it is an established model to visualize responses to treatment post-injury. The results demonstrated the role and mechanism of om-hASCexos in accelerating wound healing. The results provide an understanding of the mechanisms occurring in internal injuries, wherein the omentum promotes healing and reduces inflammation in the organs, such as spleen or liver lacerations. Our laboratory has previously published results of sc-hASCexos promoting repair and healing in mice via topical [16], intranasal [63], and intravenous [64] delivery for multiple injury models, including dermal and traumatic brain injury. Thus, om-hASCexos too can be delivered intravenously or via targeted injections in a clinical setting.

The limitations of this study include the duration of the in vivo wound model. This will be extended to about 28 days (full wound closure) in a future study. Secondly, it would be interesting to see the effects of om-hASCexos in an internal injury model, such ulcerative colitis or ruptured ovaries, which we can undertake in the future. Here, we established that the cargo of om-hASCexos is distinct from other sources of exosomes, has a robust efficacy to repair wounds, and determined the pathways and genes affected by hASCexos. Additionally, this study was performed using human-derived exosomes in a rat model. A rat model was chosen for this study due to their similarities to human physiology, along with the abundant information and resources available compared to other model organisms. It is possible that there are species-specific factors involved that would result in different responses to exosomes in rats compared to humans. Furthermore, since the exosome species of origin is different from the species receiving the exosomes, the response may not be as potent due to this cross-species interaction. This may also explain the differences in in vitro and in vivo gene expression, as in vitro studies were performed using a human cell line. Future studies may investigate human wounds treated with om-hASCexos. The in vivo experiments were only evaluated at day 7 post-wounding, which is a snapshot at this timepoint. At 7 days post-wounding, genes such as *MMP9*, *TGF-β*, and *TNFα* have either higher or equal expression in wounds treated with om-hASCexos compared to the control wounds. It is likely that these levels are changing over time as the wounds progress through the healing process. Future studies will investigate the changes that occur over time by performing a timepoint study in which multiple times post-wounding are investigated until complete wound closure is observed.

## 5. Conclusions

In summary, we evaluated protein cargo differences between om-hASCexos and sc-hASCexos. This is the first report of a proteomic analysis of exosomes derived from the omental adipose depot. We found that seven proteins had a greater enrichment in om-hASCexos compared to sc-hASCexos, while nine proteins had greater expression in sc-hASCexos compared to om-hASCexos, which reveals differences in the cargos of the exosomes originating from these two separate adipose depots. Additionally, our data show that exosomes derived from the omental adipose depot are enriched in the lncRNAs *GAS5* and *MALAT1.* There is a greater enrichment of *GAS5* and decreased enrichment of *MALAT1* in om-hASCexos compared to sc-hASCexos. This further cements the idea of cargo differences between exosomes derived from different adipose depots. Overall, we demonstrate that the treatment with om-hASCexos promoted healing both in vitro and in vivo. Our in vitro studies showed that the om-hASCexo treatment reduced cytotoxicity and improved mitochondrial function under oxidative stress and reduced inflammatory markers during LPS exposure. Histology staining of in vivo wound tissues after 7 days of healing revealed a higher microvessel density and increased collagen in wounds treated with om-hASCexos. Finally, RNAseq of wound tissue indicates that genes and pathways associated with apoptosis, cell differentiation, and immune response were downregulated in wounds treated with om-hASCexos compared to the control wounds, while those associated with fatty acid and lipid biosynthesis were upregulated.

Overall, our results demonstrate that the exosomes contained in the secretome of the omental depot are central to the healing of internal injuries, due to their ability to significantly reduce inflammation and oxidative stress while promoting cell migration and proliferation. The study establishes the distinct and unique cargo of hASCexos derived from the omental adipose depot, thus expanding the comprehension of the role of the omental adipose depot in humans. At a broader level, this knowledge can be applied to understanding how the omentum responds to insults such as high blood pressure, internal lacerations of the liver and spleen, or hyperglycemia.

## 6. Patents

Provisional patent No. 18/301,093 (NAP). No financial conflict, as it is not yet licensed.

## Figures and Tables

**Table 1:** qPCR primers used in in vitro and in vivo experiments.

Primer	Sense	AntiSense
Human IL6	AGACAGCCACTCACCTCTTCAG	TTCTGCCAGTGCCTCTTTGCTG
Human IL1-β	CCACAGACCTTCCAGGAGAATG	GTGCAGTTCAGTGATCGTACAGG
Human TGFβ	CCCATGCCGCCCTCCGGGCTGC	TCAGCTGCACTTGCAGGAGC
Human MMP9	GTGCTGGGCTGCTGCTTTGCTG	GTCGCCCTCAAAGGTTTGGAAT
Human TNFα	TCTTCTCCTTCCTGATCGTGG	TGCCTGGGCCAGAGGGCTGA
Human GAPDH	GATCATCAGCAATGCCTCCT	TGTGGTCATGAGTCCTTCCA
Rat Malat1	GGTTACCAGCCCAAACCTCA	GCATCAAGGTGAGGGGTGAA
Rat Gas5	CTGTGATGGGACATCTGGTGG	TCCCATTTTCTGGCTTCCCAT
Rat MMP9	AGGCGCCGTGGTCCCCACTTACTT	GCAGGGTTTGCCGTCTCCGTTGCC
Rat TGF-β	GCAACAACGCAATCTSTGAC	CCTGTATTCCGTCTCCTT
Rat BCL2	ATCGCTCTGTGTGGATGACTGAGTAC	AGAGACAGCCAGGAGAAATCAAAC
Rat IL6	TCCTACCCCAACTTCCAATGCTC	TTGGATGGTCTTGGTCCTTAGCC
Rat Col1	AGGGAACAACTGATGGTGCTACTG	GGACTGCTGTGCCAAAATAAGAGA
Rat Col3	AGGGAACAACTGATGCTGCTACTG	GGACTGCTGTGCCAAAATAAGAGA
Rat IL1-β	CACCTCTCAAGCAGAGCACAG	GGGTTCCATGGTGAAGTCAAC
Rat GAPDH	GGCAAGTTCAATGGCACAGT	TGGTGAAGACGCCAGTAGACTC

**Table 2:** Automated Western Blot antibodies.

Antibody	Source	Cat #
Alix	Cell Signaling Technology (CST) (Danvers, MA, USA)	92880T
CD9	Millipore Sigma (Burlington, MA, USA)	CBL162
CD69	Abcam (Cambridge, UK)	Ab216130
TSG101	Abcam (Cambridge, UK)	Ab125011
Secondary HRP for rabbit	Bio-Rad (Hercules, CA, USA)	1706515
Secondary HRP for mouse	Invitrogen (Waltham, MA, USA)	62-6520

**Table 3:** Top 20 significantly expressed proteins in om-hASCexos.

Protein	Gene	Log LFQ Intensity	STD Dev
Actin, cytoplasmic 2	ACTG1	25.99	0.18
Fibronectin	FN1	25.99	0.03
Collagen alpha-1(I) chain	COL1A1	25.73	0.26
Thrombospondin-1	THBS1	24.86	0.29
Phospholipid transfer protein	PLTP	24.82	0.11
Decorin	DCN	24.57	0.05
Glia-derived nexin	SERPINE2	24.51	0.21
Thrombospondin-4	THBS4	24.51	0.16
Collagen alpha-2(I) chain	COL1A2	24.48	0.14
Pentraxin-related protein PTX3	PTX3	24.12	0.06
Alpha-2-macroglobulin	A2M	23.76	0.75
Heat shock protein HSP 90-beta	HSP90AB1	23.46	0.02
Periostin	POSTN	23.06	0.23
Glyceraldehyde-3-phosphate dehydrogenase	GAPDH	22.71	0.10
Complement C3	C3	22.68	0.93
Alpha-enolase	ENO1	21.87	0.14
Fibulin-1	FBLN1	21.85	0.19
Filamin-A	FLNA	21.72	0.33
Heat shock cognate 71kDa protein	HSPA8	21.65	0.59
Metalloproteinase inhibitor 2	TIMP2	21.60	0.61
Galectin-3-binding protein	LGALS3BP	19.89	0.63

**Table 4:** Four GO pathway categories with included pathways.

Group Name	Included Pathways
Cell Development/Differentiation	Cell differentiation Cell development Tissue morphogenesis
Apoptosis	Apoptotic process Cell death Programmed cell death Regulation of apoptotic process Regulation of programmed cell death
Immune Response	Immune system process Immune response Cytokine production
Fatty acid and Lipid Biosynthesis	Lipid metabolic process Lipid biosynthetic process Fatty acid metabolic process Fatty acid catabolic process Cellular lipid metabolic process

## Data Availability

The raw data of the RNAseq are uploaded as supplemental data.

## Supplementary Materials

The following supporting information can be downloaded at https://www.mdpi.com/article/10.3390/biology14111509/s1. Files S1 and S2: Publication licenses from BioRender; File S3 Raw RNAseq count data. File S4: Original automated Western Blot gel image using JESS (ProteinSimple).

## Abbreviations

The following abbreviations are used in this manuscript:

EV	Extracellular Vesicles
hASC	Human Adipose Stem Cells
exo	Exosome
om	Omental
sc	Subcutaneous
GO	Gene Ontology
GSEA	Gene Set Enrichment Analysis
ORA	Over-Representation Analysis
BP	Biological Processes
DEG	Differentially Expressed Genes
TEM	Transmission Electron Microscopy
