# Supplementary figures and images for: The Human Omental Adipose Depot Mitigates Inflammation, Immune Response, and Oxidative Stress Pathways in Response to Injury via Its Secretome
Source: Biology (Basel). 2025 Oct 28;14(11):1509. doi: 10.3390/biology14111509 (PMC12650205; doi:10.3390/biology14111509)

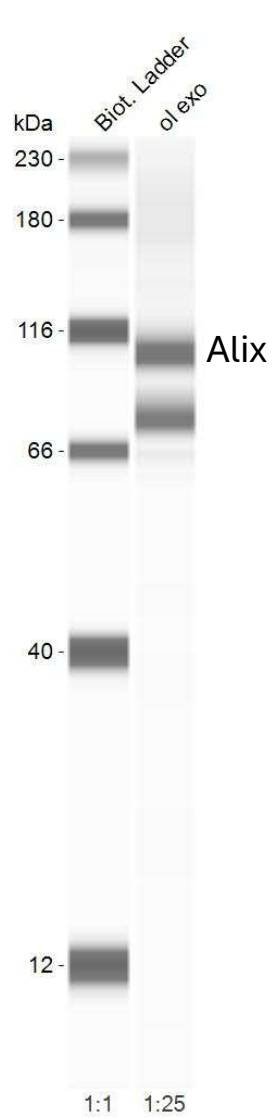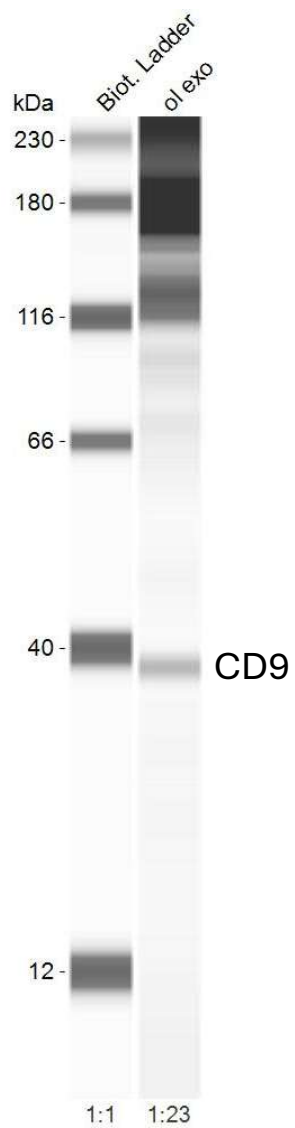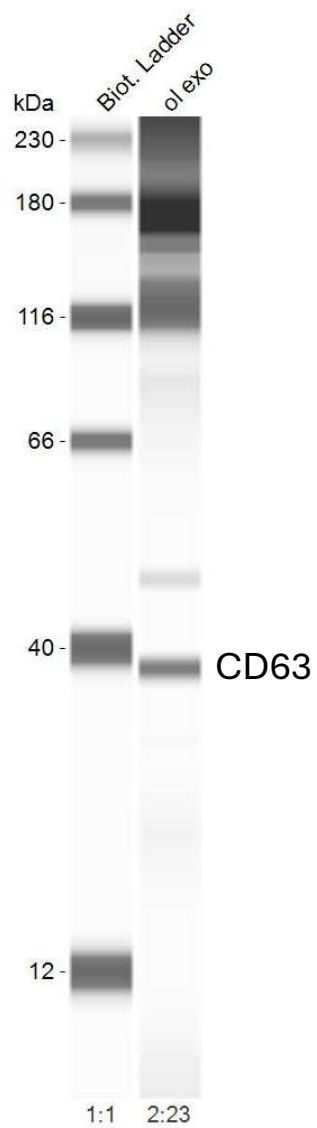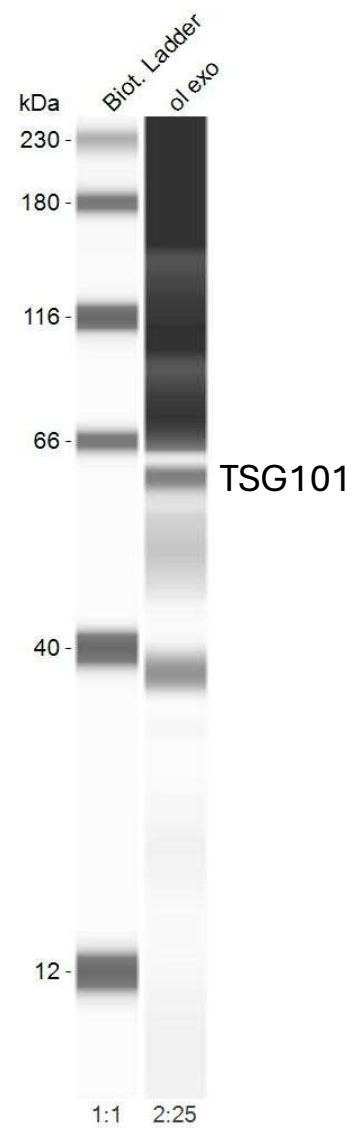

Supplement: Supplementary file 1 [file biology-14-01509-s001.zip › Supplemental S4 om-hASCexo full blots 102725.pdf]
